# Interoceptive Brain Processing Influences Moral Decision Making

**DOI:** 10.1002/hbm.70108

**Published:** 2024-12-25

**Authors:** Shengbin Cui, Tamami Nakano

**Affiliations:** ^1^ Graduate School of Medicine Osaka University Osaka Japan; ^2^ Graduate School of Information Science and Technology Osaka University Osaka Japan; ^3^ Center for Information and Neural Networks (CiNet) National Institute of Information and Communication Technology Osaka Japan

**Keywords:** alexithymia, EEG, heartbeat‐evoked potential, interoception, moral decision‐making

## Abstract

Not harming others is widely regarded as a fundamental tenet of human morality. Harm aversion based on the consequences of an action is called utilitarianism while focusing on the action itself is associated with deontology. This study investigated how interoceptive processing affects the neural processing of utilitarian and deontological moral decision‐making. The study utilized the heartbeat‐evoked potential (HEP), an averaged electrophysiological component from electroencephalogram (EEG) to gauge cardiac interoceptive processing. Twenty‐seven participants were asked to make utilitarian and deontological decisions for personal and impersonal moral dilemmas (18 for each) with direct and indirect harm actions, respectively, while their EEG and electrocardiogram were being recorded. We found no difference in HEPs between personal and impersonal moral dilemmas. In contrast, differential HEPs were observed between utilitarian and deontological moral decision‐making, regardless of type of dilemmas. Significant differences were observed over centro‐posterior electrodes between 110 and 172 milliseconds after R‐peaks during the Scenario Phase, and over right fronto‐temporal electrodes between 314 and 404 milliseconds after R‐peaks in the Decision Phase. We confirmed that these differences in HEP amplitude between deontological and utilitarian decisions did not stem from cardiac artifacts. These findings reveal that the brain utilizes interoceptive information to make subsequent moral decisions.

## Graphical abstract

The differential Heartbeat Evoked Potentials (HEPs) were observed between utilitarian and deontological moral decision‐making during both the scenario reading phase and the decision‐making phases. These findings suggest that the brain utilizes interoceptive information to make subsequent moral decisions.

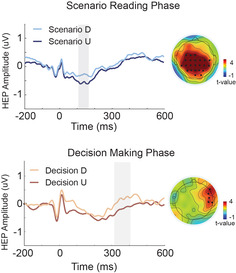

## 1 Introduction

Not harming others is considered the most fundamental principle of human morality (Cushman, Young, and Hauser 2006; Gray, Young, and Waytz 2012; Haidt 2007), and it has been confirmed that harm aversion plays a role in making moral decisions (Blair 1995; Crockett 2013; Cushman et al. 2012). The perspective taken in terms of the outcomes of an action against harm aversion is called utilitarianism (Mill 1863), whereas the one in terms of the reactions to an action per se is called deontology (Kant 1959). Moral psychology utilizes hypothetical moral dilemmas to investigate the intense debate between the common good and individual rights that are derived from the two moral perspectives (Greene et al. 2004; Greene et al. 2001; Shenhav and Greene 2014; Singer 1979). In the classic trolly problem, people were asked whether they would be willing to flip a switch to divert a running train onto another track, where it would kill one worker but save five workers on the original track (Thomson and Parent 1986). People with a utilitarian perspective will endorse turning the switch, whereas people with a deontological preference will endorse not flipping the switch.

The pattern of decisions made when confronting moral dilemmas was interpreted through the lens of the dual‐process theory of moral judgment (Kahneman 2003), as further explored by Greene et al. (2004), Greene et al. (2001), and Shenhav and Greene (2014). While outcome‐evoked harm aversion, or utilitarian moral decision, has been said to result from cognitive computation mediated by the dorsolateral prefrontal cortex and inferior parietal lobes (Greene et al. 2004), the action‐triggered harm aversion, or deontological moral decision, has derived from emotional responses mediated by brain structures such as amygdala (van Honk et al. 2022). For example, when participants experience time pressure while making moral judgments, they tend to choose deontological options due to the involvement of immediate emotions, which cannot be overridden by cognitive control (Suter and Hertwig 2011). Additionally, the valence framing effect (lives saved or lives lost) reduces the proportion of utilitarian choices in the moral judgments (Palmiotti et al. 2020). Consistently, an electrophysiological study reported that moral dilemmas elicit an aversive response in the early phase of decision making (Sarlo et al. 2012). These results suggest that moral judgments are determined by a balance between the dual processes of intuitive emotion and cognitive manipulation.

Furthermore, researchers have found that the type of harms (direct or indirect) plays a significant role in making moral decisions (Greene et al. 2009; Greene et al. 2004; Greene et al. 2001). The trolly problem mentioned above is an example of impersonal moral dilemmas (with indirect harms), the footbridge problem in which whether to push a large man off a bridge to his death to stop a trolly from running over five workers is an example of personal version (direct harms). One research has found that the default mode network (DMN) and amygdala increased activity in personal moral dilemmas, whereas the dorsolateral prefrontal cortex demonstrated heightened activity in impersonal versions (Greene et al. 2001). These results suggest that cortical processing related to abstract reasoning leads to utilitarian decisions in impersonal moral dilemmas, and that cortical processing related to mentalization and emotion inclines towards deontological decisions in personal moral dilemmas. Previous studies (Koenigs et al. 2007; Moretto et al. 2010) have reported that patients with damage to the ventromedial prefrontal cortex (vmPFC), which is crucial for generating social emotions (Beer et al. 2003; Damasio 2003; Damasio, Tranel, and Damasio 1990), tend to make more utilitarian decisions in personal moral dilemmas. Similarly, individuals exhibiting psychopathic traits, characterized by reduced empathy and lowered emotional responsiveness, are also inclined to make more utilitarian decisions in personal dilemmas (Bartels and Pizarro 2011; Ng et al. 2024; Pletti et al. 2017; Tassy et al. 2013). These findings indicate that emotional functioning has a strong influence on moral decision‐making especially in personal moral dilemmas.

Considering the significance of afferent feedback from bodily responses in constructing emotions and decision‐making, as proposed by the somatic marker hypothesis (Bechara and Damasio 2005; Damasio 2005), interoceptive processing might also influence moral decision‐making, especially in personal moral dilemmas. Interoception is defined as the ability to perceive information representing internal bodily states (Craig 2002, 2003, 2009). Previous research has shown that action‐based harm aversion was derived from visceral reactions of the autonomic nervous system (Blair 1995), and another research also found that immediately evoked physiological reactions by harmful actions lead people to evaluate such actions as morally unacceptable regardless of consequences (Cushman et al. 2012). Also, previous studies have identified the insular cortex and anterior cingulate cortex (ACC) as key areas for interoceptive processing (Couto et al. 2015; Park et al. 2018; Park et al. 2014; Pollatos, Kirsch, and Schandry 2005) as well as integration with emotional processing (Adolfi et al. 2017; Kim et al. 2019). These regions have also been demonstrated to be involved in decision‐making (Thielscher and Pessoa 2007).

Given these facts, the interaction and integration of emotions with interoceptive processing may play an important role in making moral decisions rather than emotional processing alone. This possibility is supported by a previous study reporting that the skin conductance response, an index of sympathetic nerve activity, increases before making utilitarian choices in personal dilemmas (Moretto et al. 2010). Another study showed that people with lower heart rate variability (HRV) tend to make utilitarian decisions, indicating that the functional integration of neural and visceral systems might constrain utilitarian preference (Park, Kappes, et al. 2016). Furthermore, people with alexithymia, a personal trait that is characterized by difficulty in identifying, conveying, and describing one's feelings and decreased empathy (Sifneos 1973), tend to make utilitarian judgments due to their reduced empathy (Patil and Silani 2014). Individuals with autism spectrum disorders (ASD) also tend to make more utilitarian judgments in personal moral dilemmas (Gleichgerrcht et al. 2013). A subsequent study revealed that the presence of alexithymia trait in these individuals is linked to this increased utilitarian bias (Patil et al. 2016). These previous studies focused exclusively on the relationship between visceral responses and moral decision‐making. They highlight whether moral emotions, in response to moral dilemmas, induce bodily responses that consequently affect moral decisions, or if moral emotions directly impact cortical interoceptive processing, leading to utilitarian or deontological decisions. However, no study has yet directly investigated the relationship between cortical interoceptive processing and moral decision‐making. To fill this significant gap, the present study explores how interoceptive processing influences moral decisions in personal and impersonal scenarios.

**FIGURE 1:**
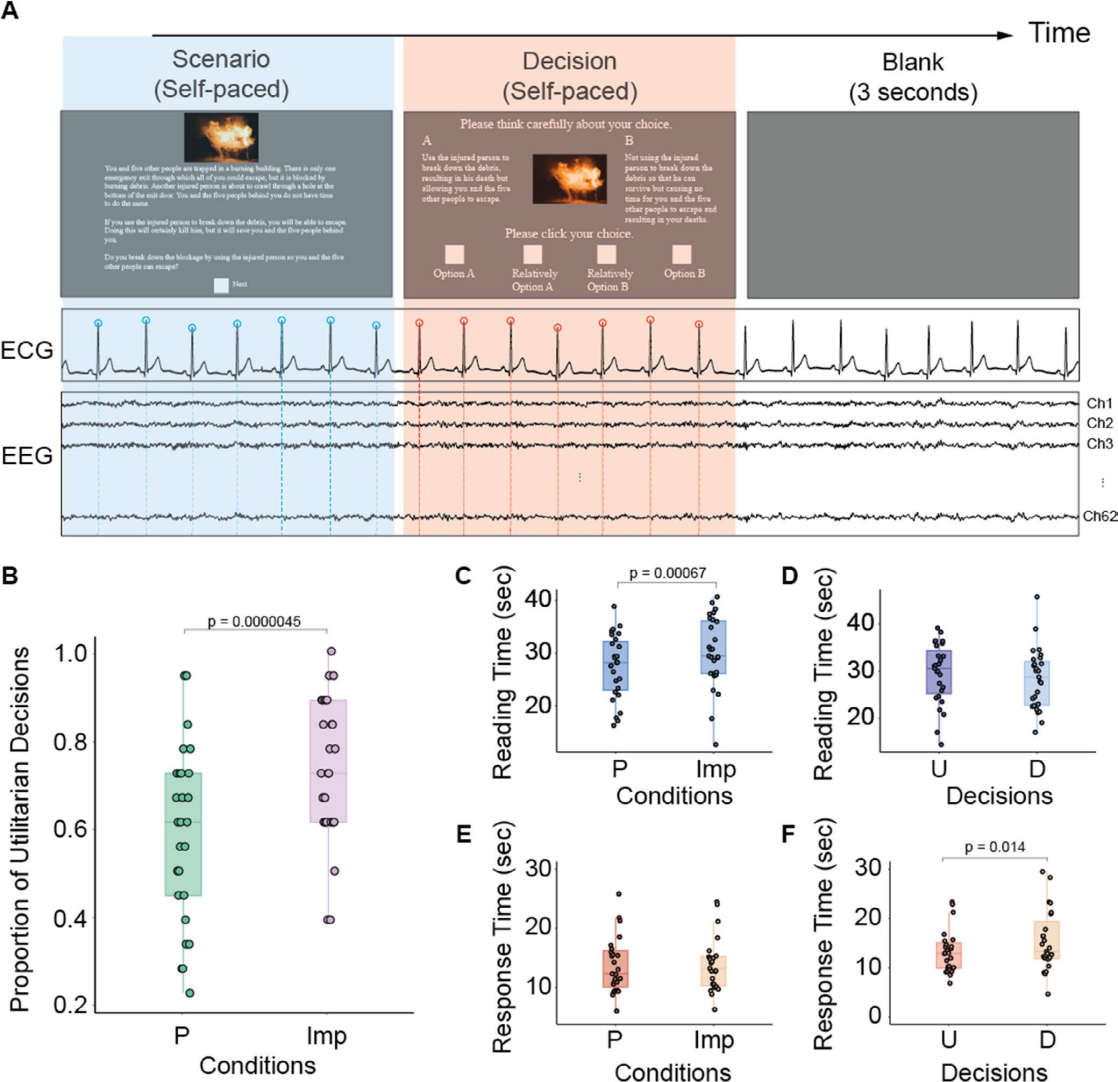
Experimental paradigm and behavioral results. (A) The experiment began with a 1.5‐s fixation period, followed by the presentation of a moral scenario and its corresponding image. Participants read the scenario at their own pace and then pressed the “Next” button to view two options. They were instructed to select one of four choices. During the Scenario (blue shaded) and Decision (red shaded) phases, electroencephalogram (EEG) and electrocardiogram (ECG) signals were recorded simultaneously. Neural responses to heartbeats were analyzed separately for the Scenario and Decision phases by averaging EEG signals time‐locked to the R‐peaks in ECG signals (indicated by blue and red circles) for personal and impersonal conditions and for utilitarian and deontological decisions, respectively. (B) The proportion of utilitarian decisions in the personal and impersonal conditions. (C) The average reading time for moral scenarios in personal and impersonal conditions. (D) The average reading time for moral scenarios in utilitarian and deontological decisions. (E) The average response times for decision‐making in personal and impersonal conditions. (F) The average response times for making utilitarian and deontological decisions. D: deontology; Imp: impersonal; P: personal; U: utilitarianism.

Previous research reported that individual differences in interoceptive accuracy based on heart rate estimation do not affect moral judgment (Tamura, Kobayashi, and Ohira 2022). Therefore, this study focuses on examining the relationship between objective interoceptive processing in the brain and moral judgments, rather than subjective interoceptive accuracy. Specifically, we concentrated on the heartbeat‐evoked potential (HEP) as a neurophysiological marker for cardiac interoceptive processing (Couto et al. 2015; Pollatos, Kirsch, and Schandry 2005). HEP is an electrophysiological component obtained by averaging time‐locked signals to heartbeats (Schandry and Montoya 1996). It typically manifests approximately 200–600 ms after the R‐peak onset in the electrocardiogram (ECG) over the somatosensory, insula, vmPFC, posterior cingulate cortex and parietal lobules (Al et al. 2021; Azzalini et al. 2021; Azzalini, Rebollo, and Tallon‐Baudry 2019; Babo‐Rebelo, Richter, and Tallon‐Baudry 2016; Babo‐Rebelo et al. 2016; Couto et al. 2015; Kern et al. 2013; Park et al. 2014). Time‐frequency decomposition analysis further revealed an increase in intertrial coherence (ITC) within 4–10 Hz range without any changes in spectral power (Park et al. 2018). This suggests that the phase resetting of neural activity contributes to the HEP generation. Thus, this study presented scenarios that elicited either personal or impersonal dilemmas and instructed participants to select between utilitarian and deontological decisions (Figure 1A). We then compared the HEPs recorded during these decision‐making processes for the two types of scenarios and two moral perspectives. We hypothesized that HEP amplitude would be greater in personal dilemmas than in impersonal ones and would be greater in deontological decisions than in utilitarian decisions due to enhanced interoceptive processing evoked by negative emotions.

## 2 Materials and Methods

### 2.1 Participants

Twenty‐seven Japanese students (15 women; mean age = 22.11 ± 2.10 years) from Osaka University participated in this experiment. The sample size was estimated using G*Power 3.1. We estimated a Cohen's *d* effect size of 0.6, with a significance level set at alpha = 0.05, and the power (1 – beta) at 0.85. The total estimated sample size was 27.

In the application stage for participants, individuals exhibiting traits associated with social impairments were excluded based on their scores from the Autism Spectrum Quotient 10 (AQ10) for adults (Allison, Auyeung, and Baron‐Cohen 2012). Previous research has suggested that individuals with reduced empathy have difficulty experiencing affect when facing moral dilemmas (Bzdok et al. 2012; Moretto et al. 2010; Patil and Silani 2014) and that ASD is characterized by impaired empathetic ability (Kanner 1968; Schnitzler and Fuchs 2023; Song et al. 2019). A previous study validated a score above 6 is an indication of high levels of autistic traits (Booth et al. 2013). Therefore, applicants scoring higher than 6 points on the AQ10 were excluded from this study.

The experimental protocol was approved by the review board of Osaka University (L028), and all procedures adhered to the Declaration of Helsinki. Written consent was obtained from all the participants before their participation, and they were compensated with 6000 Japanese Yen via bank transfer for their involvement.

### 2.2 Moral Dilemma Scenarios

The moral dilemma scenarios used in this study were adapted from a previous study by Christensen et al. (2014), which validated the set of moral dilemma scenarios fine‐tuned across several dimensions including personal force, benefic recipient, evitability, and intention, as well as methodological aspects such as word count, expression style, and question formats. We selected 18 pairs of moral dilemmas in terms of personal force (personal and impersonal). Since a Japanese version was not available, we translated all the scenarios into Japanese. The accuracy of the translated version was verified through behavioral tests with four evaluators. The average word count was 317 words in the personal condition and 325 words in the impersonal condition, showing no significant statistical difference between the two. All scenarios used in this study are included in the Supporting Information.

### 2.3 Procedure

After obtaining written consent, participants were asked to fill in the validated Japanese version of the Toronto Alexithymia Scale‐20 (TAS‐20) questionnaire (Bagby, Taylor, and Parker 1994; Komaki et al. 2003) to assess trait alexithymia. The TAS‐20 is a 20‐item scale that has been said to be the best current measure for assessing alexithymia (Timoney and Holder 2013). Higher scores indicate a higher level of alexithymia.

Next, participants were asked to proceed to the behavioral task. The experiment was designed and controlled using PsychoPy (Peirce et al. 2019) and displayed on a 23‐in. monitor (1920 × 1080 pixels, BenQ). Each participant completed two sessions with 18 trials per session. In these sessions, 18 pairs of moral scenarios were presented in a pseudo‐randomized order. In each session, either the personal or impersonal scenario of each pair was presented to ensure that no session presented a scenario from the same pair.

Each trial began with a white fixation on the screen's center for 1.5 s (Fixation Phase). Subsequently, a moral dilemma scenario and a context‐related image were displayed (Scenario Phase in Figure 1A). The scenario appeared in an 1100 × 800 pixels frame at the bottom of the screen, whereas the corresponding image was displayed above it, sized at 400 × 225 pixels. Participants were instructed to read the scenario at their own pace and click on a button to proceed to the next screen. They were then instructed to select either Option A or Option B. Based on findings from a previous study by Guzman et al. (2022), which reported that individuals often make compromises between conflicting values rather than adopting extreme positions, this study asked participants to make their selections using a 4‐point scale: Choice 1 for Option A (utilitarian extreme), Choice 4 for Option B (deontological extreme), and Choices 2 and 3 for their compromised actions toward Option A and Option B, respectively (Decision Phase). After making their decisions, participants took a 3‐s break (Blank Phase).

### 2.4 Physiological Recording and Analysis

Continuous EEG signals were recorded from all the participants during the behavioral task using a 64‐channel active‐electrode EEG system (ActiCAP; BrainProduct, Germany) at a sampling rate of 500 Hz (Figure 1A). An online filter between 0.1 and 70 Hz was applied during recording. The reference electrodes were placed on the earlobes of both ears, and their average was used as a reference. Continuous cardiac signals were collected using bipolar ECG electrodes placed at the top of the right shoulder and the bottom of the left side of the abdomen.

EEG preprocessing was performed using the EEGLAB toolbox (Delorme and Makeig 2004) and MATLAB2021b (MathWorks, USA). The recorded continuous EEG data were filtered offline between 1 and 30 Hz. We selected these frequency bands based on findings from a previous study, which reported that HEPs consist of low frequency components under 20 Hz (Park et al. 2018). Malfunctioning electrodes were interpolated using the average of the surrounding electrodes. Independent components indicative of eye movements, blink artifacts, and cardiac artifacts were visually identified and removed using independent component analysis (independent components removed: M = 3.63, SD = 1.04) (Delorme, Sejnowski, and Makeig 2007; Makeig et al. 2004).

The HEPs were calculated based on EEG signals locked to the R‐peaks from the ECG data (Montoya, Schandry, and Muller 1993; Schandry and Montoya 1996; Schandry, Sparrer, and Weitkunat 1986). Prior to this, a 0.1‐Hz high‐pass filter was applied offline to the continuous ECG data. The R‐peaks were identified using MATLAB's peakfinder function and were extracted from the time at which the moral scenarios were shown (Scenario Phase in Figure 1A) and the time at which the options were displayed until the participants made a decision (Decision Phase in Figure 1A). EEG signals were then segmented into epochs ranging from −200 to 600 ms relative to each R‐peak onset. Noisy epochs that are either higher than 40 μV or lower than −40 μV were rejected after baseline correction relative to −70 to −40 ms from the R‐peaks. Additionally, every epoch was visually inspected to ensure artifact‐free data. Next, epochs within the phase where participants read the scenario at their own pace (Scenario Phase), and epochs within the Decision Phase from when options were presented to when the selection button was pressed, were averaged for each condition. In the Scenario Phase, the average number of discarded epochs was 29.12 (SD = 57.83) for the personal condition and 30.12 (SD = 63.93) for the impersonal condition. For the utilitarian and deontological decisions, the averages were 38.96 (SD = 80.33) and 20.28 (SD = 42.75), respectively. In the Decision phase, the average number of discarded epochs was 24.74 (SD = 52.48) in the personal condition and 25.29 (SD = 51.75) in the impersonal condition. For the utilitarian and deontological choices, the averages were 30.26 (SD = 62.90) and 20.07 (SD = 45.37), respectively.

After artifact correction, the average number of epochs was 568.08 ± 139.085 epochs for the personal condition, 628.08 ± 160.87 for the impersonal condition, 775.44 ± 254.86 for utilitarian decisions, and 420.72 ± 228.80 for deontological decisions in the Scenario Phase. In the Decision Phase, the average number of epochs were 281.36 ± 129.33 epochs for the personal condition, 287 ± 126.88 epochs for the impersonal condition, 348.24 ± 190.46 epochs for utilitarian decisions, and 220.48 ± 145.72 for deontological decisions.

Time‐frequency analysis of the HEPs was computed using the Fourier transform for frequencies ranging from 1 to 30 Hz for all epochs for utilitarian and deontological decisions in both Scenario and Decision phases in each participant.

### 2.5 Statistical Analyzes

A cluster‐based permutation *t* test was used to identify significant differences in the averaged HEPs between personal and impersonal conditions (Maris and Oostenveld 2007). *T* values exceeding a threshold of *p* < 0.05 were grouped based on temporal adjacency in each channel. Cluster‐level statistics were applied to each cluster and the sum of the *t* values of the samples. To control for the Type 1 error rate, the maximum cluster‐level statistics under the null hypothesis were evaluated. Condition labels were randomly shuffled 1000 times to estimate the distribution of the maximal cluster‐level statistics by chance. For multiple testing corrections across 62 channels, A Monte Carlo *p* value (*p* < 0.05) was obtained. This value corresponds to the proportion of elements in the distribution of the randomized maximal cluster‐level statistics, exceeding the observed maximum or minimum original cluster‐level test statistics. This procedure was conducted at the electrode level over a time window of 0–600 ms after R‐peak onset. In addition, the same statistics were also applied to the averaged HEPs between utilitarian and deontological decisions.

To confirm whether the observed differential HEPs were attributable to the timing of R‐peaks, we generated surrogate HEP data. These data preserved the original intervals and variability of R‐peaks but lost timing information (Park and Blanke 2019). The onsets of all original R‐peaks were randomly shifted within a range of −500 to 500 ms. We created 1000 sets of these surrogate R‐peaks and calculated the HEPs for each decision during both the Scenario and Decision phases for each participant. We then computed the average and standard deviation of the HEP differences across these surrogate datasets.

Next, the difference in the proportion of utilitarian decisions between personal and impersonal conditions was examined with paired *t* test. The differences in the reading times and response times between personal and impersonal conditions and between utilitarian and deontological decisions were also investigated with the same statistics. Repeated‐measures ANOVA was used to examine the effect of the moral perspectives (utilitarianism, deontology) and trade‐offs (extreme, compromised). Then, the correlation between the averaged HEP amplitude difference from the electrodes with a significant difference and TAS‐20 scores, reading times, response times, and heart rate differences were calculated and the correlation between TAS‐20 scores and the proportion of utilitarian decisions was also calculated.

Finally, we conducted another cluster‐based permutation *t* tests on the averaged time‐frequency results over the selected channels of interest during the Scenario and Decision phases between utilitarian and deontological decisions. Clusters were defined as contiguous time‐frequency points where the *t*‐statistics exceeded a threshold (*p* < 0.05) to determine statistical significance. The sum of *t* values within each cluster was computed to generate cluster‐level statistics. To control for the Type I error rate, the maximum cluster‐level statistics under the null hypothesis were evaluated. This process was repeated over 1000 permutations with randomized condition labels to create a null distribution of cluster statistics. Observed cluster statistics were then compared against this null distribution to assess significance.

## 3 Results

### 3.1 Behavioral Results

As illustrated in Figure 1B, the proportion of utilitarian decisions in the impersonal condition was significantly greater than that in the personal condition (personal: mean (M) = 0.59, standard deviation (SD) = 0.20; impersonal: M = 0.73, SD = 0.16; paired *t* test, *t*_(26)_ = −5.77, *p* = 0.0000045, Cohen's *d* = 0.81). These results align with those of previous studies (Greene et al. 2001). In comparison between the extreme and compromised choices, the participants tend to opt the compromised choice rather than the extreme one for the personal condition (compromised M = 0.62, SD = 0.23). On the other hand, the mean proportion of compromised choice for the impersonal condition was 0.54 (SD = 0.24). The significant difference was detected in the ratio of compromised choice between the personal and impersonal conditions (paired *t* test, *t*_(26)_ = 2.54, *p* = 0.02, Cohen's *d* = 0.49). Also, no correlation between TAS‐20 scores and the proportion of utilitarian decisions was observed (*r* = −0.20, *p* = 0.35).

The mean reading time for the moral scenarios in the impersonal condition was significantly longer than in the personal condition (Figure 1C, personal: M = 27.40 SD = 6.048; impersonal: M = 30.12, SD = 6.74, paired *t* test, *t*_(26)_ = −3.86, *p* = 0.00067, Cohen's *d* = 0.42), In contrast, no significant difference was observed between utilitarian and deontological decisions (Figure 1D, utilitarianism: M = 29.51, SD = 6.42; deontology: M = 28.32, SD = 6.48; paired *t* test, *t*_(26)_ = 1.0081, *p* = 0.32, Cohen's *d* = 0.18). As depicted in Figure 1E, no significant difference in response times during the Decision Phase was observed between the personal and impersonal conditions (personal: M = 14.54, SD = 6.91; impersonal: M = 14.61, SD = 6.40; paired *t* test, *t*_(26)_ = −0.21, *p* = 0.84). These results suggest that individuals tend to opt for deontological decisions to utilitarian decisions when confronted with personal moral dilemmas despite the similarity in response times. However, a significant difference was observed in response times between utilitarian and deontological decisions (Figure 1F; utilitarianism: M = 14.08, SD = 6.54; deontology: M = 16.08, SD = 7.54; paired *t* test, *t*_(26)_ = −2.62, *p* = 0.014, Cohen's *d* = 0.28), indicating that participants tend to spend longer time in making deontological decisions, regardless of the type of harms.

### 3.2 HEP Results

Next, HEPs averaged over EEG signals time‐locked to the R‐peaks of the ECG were separately compared between conditions and decisions during the Scenario and Decision phases. Two participants were excluded from all subsequent HEP analyzes because they made fewer than two deontological decisions out of 36 trials, a number too small to reliably analyze EEG waveforms. For the remaining participants, the number of deontological decisions ranged from 6 to 24 out of 36 trials, ensuring a sufficient range for robust EEG analysis.

**FIGURE 2:**
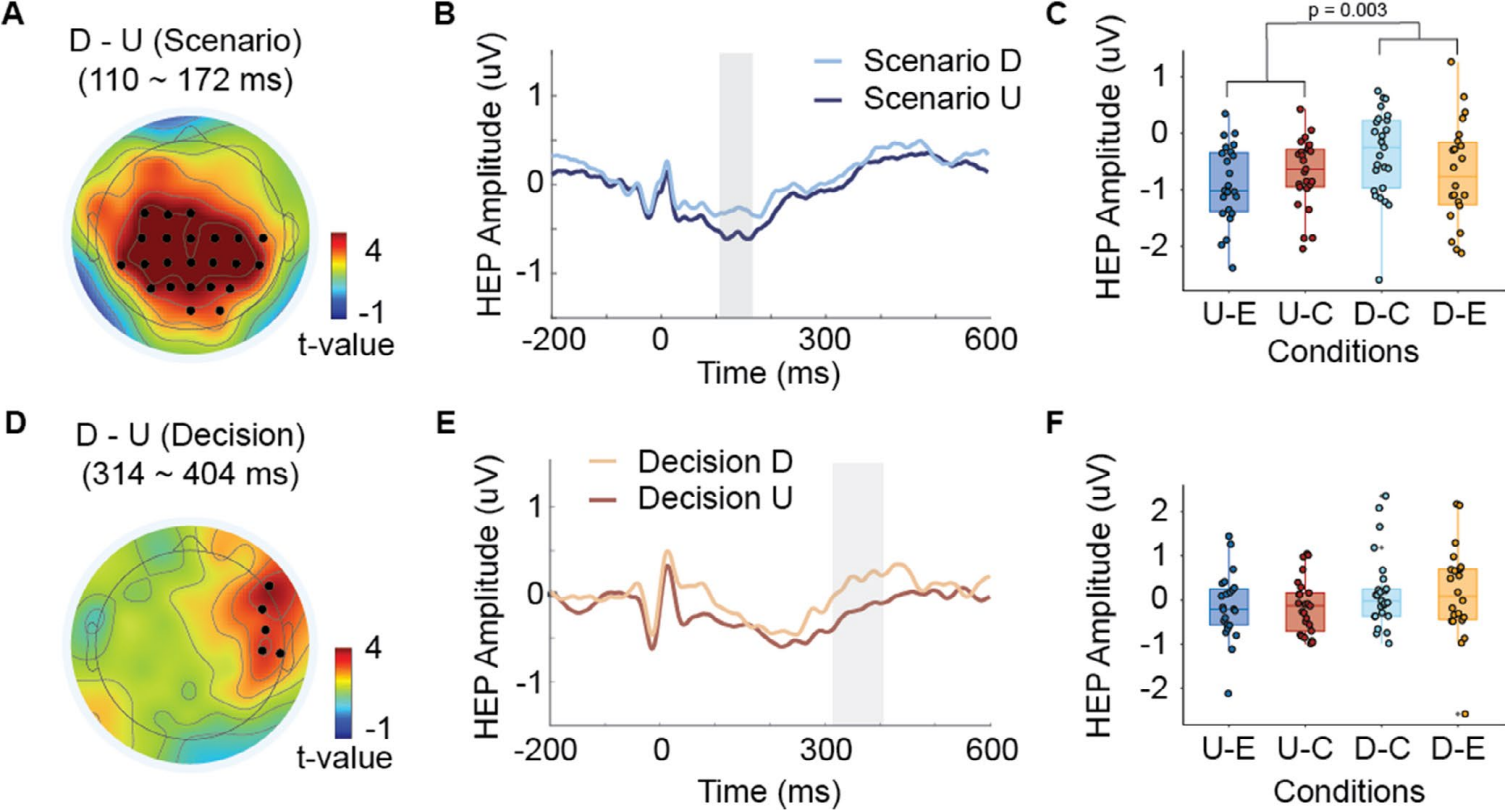
Comparison of neural responses to heartbeats between utilitarian and deontological choices in the Scenario and Decision phases. (A) Topographical map illustrating the differential heartbeat‐evoked potentials (HEP) amplitude between utilitarian and deontological decisions within 110–172 ms time window after the onset of R‐peak where significant differences were observed in the Scenario phase. Black dots represent electrodes contributing to the significant clusters. (B) Time course of averaged HEP over the electrodes with significant clusters found between utilitarian and deontological decisions in the Scenario phase, illustrating that the HEP amplitudes for deontological decisions were significantly greater than those for utilitarian decisions (indicated by the blue‐shaded area). The light blue and dark blue lines represent deontological and utilitarian decisions, respectively. (C) Mean HEP amplitudes over the electrodes with significant difference were calculated for utilitarian and deontological decisions, and extreme and compromised tendencies. (D) Topographical map illustrating the differential HEP between utilitarian and deontological decisions within 314–406 ms after R‐peak where significant differences were observed in the Decision phase. Black dots indicate electrodes with significant difference. (E) Time course of mean HEP over the significant electrodes between utilitarian and deontological decisions in the Decision phase, demonstrating that the HEP for deontological choices were significantly greater than those for utilitarian choices (red‐shaded area). The orange and red lines represent deontological and utilitarian decisions, respectively. (F) Mean HEP over the significant electrodes for utilitarian and deontological decisions, and extreme and compromised tendencies. C: compromised; D: deontology; E: extreme; U: utilitarian.

We first compared HEP amplitudes between the personal and impersonal conditions during the Scenario Phase and found no significant differences across any electrodes. In contrast, significant differences were observed between utilitarian and deontological decisions over central electrodes (FC1, C3, CP5, CP1, Pz, P3, P4, CP6, CP2, Cz, C4, FC3, C1, CP3, P1, POz, PO4, P2, CPz, CP4, C6, C2, and FCz) from 110 to 172 ms post‐R‐peak (Figure 2A,B). During the Decision Phase, no significant differences were found between the personal and impersonal conditions; however, significant differences emerged between utilitarian and deontological decisions over right frontal, central, and parietal electrodes (F8, FC6, C6, CP6, and TP8) ranging from 314 to 404 ms post‐R‐peak (Figure 2D,E). Furthermore, we analyzed the relationship between HEP modulation during the Scenario and Decision phases, and found no significant correlations (*r* = 0.17 and *p* = 0.43).

We also tested whether the observed differential HEPs derived from moral dilemmas were truly attributable to the timing of cardiac activity using surrogate shuffling methods. Our analysis found that the actual differential HEPs exceeded 2.5 standard deviations of the surrogate data distribution (*p* < 0.05) during both the Scenario and Decision phases, with significant intervals overlapping those identified via cluster analysis on the real HEPs (see Figure S1). These findings suggest that the differential HEPs reflect genuine cardiac interoceptive processing.

We further examined the effects of reading time differences in the Scenario Phase and response time differences in the Decision Phase between utilitarian and deontological decisions on HEP differences in the ROI. In either Scenario or Decision phases, no significant correlations were observed between them (Scenario, *r* = −0.07, *p* = 0.73; Decision, *r* = −0.17, *p* = 0.42). This suggests that reading time and response time differences do not necessarily influence HEP modulation.

In addition, we performed a time‐frequency analysis of the HEPs for these significant channels using the Fourier transform and compared the relative power and relative ITC between utilitarian and deontological decisions in the Scenario and Decision phases. As a result, no clusters with statistically significant differences were identified between these decisions in either the Scenario or Decision phases, as determined by the cluster‐based permutation *t* tests on the time‐frequency representations (see Figure S2).

Subsequently, we compared the average values in these channels during this time window for utilitarian and deontological decisions in both extreme and compromised options across both phases. As shown in Figure 2C, during the Scenario Phase, there was no interaction between moral perspectives (utilitarianism, deontology) and trade‐off types (extreme, compromised) (*F*_(1,19)_ = 0.024, *p* = 0.88, *η* ^*2*^ = 0.00049). However, we observed a main effect of moral perspectives (*F*_(1,19)_ = 11.86, *p* = 0.003, *η* ^*2*^ = 0.91). In contrast, as demonstrated in Figure 2F, there was no interaction between moral perspectives and trade‐offs during the Decision Phase (*F*_(1,19)_ = 0.053, *p* = 0.82, *η* ^*2*^ = 0.001) and no main effect of moral perspectives (*F*_(1,19)_ = 3.46, *p =* 0.078, *η* ^*2*^ = 0.054) (Note that we performed analysis on 20 participants because the other 5 participants did not make all 4 choices across trials).

Furthermore, there was no correlation between the difference in averaged HEP amplitude (deontology minus utilitarian) from channels with a significant difference in both phases and TAS‐20 scores (Scenario: *r* = −0.19, *p* = 0.36; Decision: *r* = −0.11, *p* = 0.61) and heart rates difference (deontology minus utilitarian) in both phases (Scenario: *r* = 0.33, *p* = 0.11; Decision: *r* = −0.22, *p* = 0.28).

### 3.3 Cardiac Activities

To consider the influence of cardiac artifacts on the HEP result (Dirlich et al. 1997), we analyzed whether the heart rates and ECG waveforms differed between two conditions and two decisions during both phases. However, no significant differences in heart rates were found between personal and impersonal moral dilemmas during the Scenario (personal: M = 71.46, SD = 11.11; impersonal: M = 71.99, SD = 11.96; paired *t* test, *t*_(24)_ = −1.28, *p* = 0.21; utilitarian: M = 72.036, SD = 12.15; deontology: M = 71.35, SD = 10.95; paired *t* test, *t*_(24)_ = 1.43, *p* = 0.17) and Decision phases (personal: M = 71.91, SD = 11.47; impersonal: M = 72.19, SD = 11.41; paired *t* test, *t*_(24)_ = −1.73, *p* = 0.097; utilitarianism: M = 72.064, SD = 11.31; deontology: M = 71.79, SD = 11.65; paired *t* test, *t*_(24)_ = 1.51, *p* = 0.15). Furthermore, no clusters of differences were observed in the average ECG waveforms between the two types of dilemmas and two moral perspectives during both phases. These results suggest that the observed differences in HEPs between utilitarianism and deontology were not attributable to cardiac artifacts (Delorme, Sejnowski, and Makeig 2007).

## 4 Discussion

This study aimed to examine whether cortical interoceptive processing is involved in decision‐making when faced with moral dilemmas, by analyzing neural responses to heartbeats. First, as expected, participants were more inclined to make utilitarian decisions in impersonal dilemmas, indicating that opting for utilitarian decisions in personal moral dilemmas involves personal moral violations. Next, neural responses to heartbeats were more pronounced in the centro‐posterior electrodes during the Scenario Phase, and in the right front‐temporal regions in the Decision Phase, when making deontological decisions compared to utilitarian decisions. Further analyzes revealed that this modulation of HEP increased during making deontological decisions, regardless of the type of harms (direct or indirect) or degree of certainty in choice.

### 4.1 HEP as an Index of Cardiac Interoceptive Processing

Intracranial neural recordings studies from epilepsy patients have shown that electrical potential changes were observed in the somatosensory and insular cortices around 300 ms after the R‐peak of the cardiac cycle (Kern et al. 2013; Park et al. 2014). This heart‐beat‐evoked potential, known as HEP, can be also detected in the scalp EEG recordings. Various studies have shown that HEP modulation is associated with attention to interoception and emotional processing (Couto et al. 2015; Gentsch et al. 2019; Marshall et al. 2018; Montoya, Schandry, and Muller 1993; Pollatos, Kirsch, and Schandry 2005). This HEP modulation in the scalp EEG was primarily observed in the frontal (Babo‐Rebelo et al. 2016; Gentsch et al. 2019; Park, Bernasconi, et al. 2016), central (Montoya, Schandry, and Muller 1993), and parietal electrodes (Babo‐Rebelo et al. 2016). Independent component analysis (ICA) identified several brain regions involved in generating the HEP: activity increased in the superior parietal area from 0 to 400 ms, in the right centro‐lateral region including insula from 100 to 500 ms, and in both the central region and the precuneus from 200 to 600 ms post‐R peak (Lee et al. 2024). Importantly, this HEP modulation is not the result of cardiac artifacts, as there were no differences in heart rates or ECG waveforms between conditions (Park and Blanke 2019). Therefore, even though the neural mechanism underlying HEP are not fully understood, the HEP modulation over extended periods post‐R peak can serve as a marker for cardiac interoceptive processing across various brain regions. The present study revealed that during the decision‐making process in moral dilemmas, HEP varies depending on whether the decisions are utilitarian or deontological. Specifically, modulations were observed over the central‐posterior region during the Scenario Phase and over the right fronto‐temporal regions during the Decision Phase. It suggests that the central‐parietal regions utilize interoceptive processing for understanding and reacting to dilemmas situations, while the right fronto‐temporal regions utilize interoceptive processing for the selection among conflicting decisions. These findings imply that cardiac interoceptive processing has an impact on guiding subsequent moral decision‐making throughout the various stages from understanding the situation to make a decision. Damasio's somatic marker theory posits that the neural representation of interoception aids in rapidly evaluating various risks for optimal decision‐making (Damasio 2005). Previous studies consistently show that fast gut feelings associated with emotional responses leads to deontological judgments (Sarlo et al. 2012; Suter and Hertwig 2011). This aligns with Green's dual‐process model of moral judgments, which posits that moral decision‐making involves the integration of intuitive emotional and cognitive processes (Greene et al. 2001). Building on these concepts, we assume that modulation in HEP reflects the internal representations of gut feelings associated with emotional responses, which guide optimal and adaptive decisions, especially in complex moral situations.

### 4.2 HEP Reflects Emotional Processing in Moral Dilemmas Regardless of the Type of Harms

It is worth noting that the HEP modulation occurs between utilitarian and deontological decisions, regardless of the type of harms (direct or indirect). Previous studies have highlighted the significant role of harm action type in moral decision‐making (Greene et al. 2009; Greene et al. 2004). Specifically, participants are inclined to make deontological decisions in personal moral dilemmas and opt for utilitarian choices in impersonal scenarios. This trend is interpreted to mean that direct harm actions in personal scenarios elicit stronger autonomic and emotional responses than indirect harms in impersonal scenarios (Greene et al. 2004; Greene et al. 2001). This interpretation is supported by neuroimaging studies showing that brain regions related to emotion are more activated in personal scenarios (Greene et al. 2009; Greene et al. 2004). In line with the previous research, this study also found that there was a tendency to make different choices depending on the type of scenario. However, we found no significant difference in HEP amplitudes between personal and impersonal scenarios. Instead, regardless of harm action type, the degree of HEP modulation per se significantly influences moral decision‐making. These findings suggest that rather than the type of harms, the degree of interoceptive and emotional processing within the scenario influences the choice of moral decision‐making.

### 4.3 Cardiac Interoceptive Processing Within Individuals Affects Moral Decision‐Making

Previous studies have explored the impact of individual differences in autonomic activity and interoceptive sensitivity on moral judgments (Park, Kappes, et al. 2016; Patil and Silani 2014; Tamura, Kobayashi, and Ohira 2022). For example, Park, Kappes, et al. (2016) found that individuals with lower heart rate variability (low activity of the sympathetic nervous system) are more likely to make utilitarian choices. Similarly, individuals with alexithymia, characterized by low interoceptive sensitivity, tend to make utilitarian judgments (Patil and Silani 2014). Diverging from these previous studies focusing on the impact of individual traits on moral decision‐making, the present study explores how interoceptive processing in the brain influences moral decision‐making on an individual level. We found that cardiac interoceptive processing fluctuated with each trial during the Scenario and Judgment phases of moral dilemmas. The magnitude of these changes varied depending on whether the subsequent choice aligned with deontological or utilitarian principles. Previous research has indicated that deontological decisions, especially in scenarios involving direct harm, are associated with negative emotional responses (Shenhav and Greene 2014). Moreover, such decisions are accompanied by increased activity in brain regions involved in emotion and theory of mind, such as the vmPFC, amygdala, and precuneus (Bzdok et al. 2012; Koenigs et al. 2007; van Honk et al. 2022). Additionally, negative emotions have been reported to coincide with heightened interoceptive processing (Chen et al. 2024; Kim et al. 2019). These findings suggest that when faced with moral dilemmas, heightened interoceptive processing may bias individuals toward deontological judgments. However, the present study only established a correlation between heartbeat‐related brain activity and immediate choice of action. Future research should investigate whether intentionally enhancing cardiac interoceptive processing may bias moral decision making toward deontology. This direction could provide further insight into the neural basis of moral judgment and its manipulation by interventions.

### 4.4 Limitations

This study focuses on brain activity related to the heartbeat observed over a long period during the process of moral decision‐making. As such, it is difficult to precisely determine how these components contribute to the various elements and their integration within moral decision‐making. The changes in HEP observed before decision‐making merely suggest that information processing related to bodily states might influence these decisions. Future research is necessary to more precisely analyze the role of cardiac processing in moral decision‐making. Developing experimental designs that can isolate individual elements within moral tasks and investigating the relationships between HEP and emotional and bodily state evaluations might deepen our understanding of the impacts of HEP on moral decision‐making.

## 5 Conclusion

In conclusion, the present study reinforces previous research emphasizing the role of emotion when making deontological decisions (Greene et al. 2001) and further extends the idea that this involves enhanced cortical interoceptive processing. This suggests that interoceptive signals are utilized when making moral decisions, which demonstrates the somatic marker hypothesis (Bechara and Damasio 2005; Damasio, Tranel, and Damasio 1991). Furthermore, interoceptive modulation within individuals plays a role in making moral decision‐making. To the best of our knowledge, this study is the first to demonstrate the involvement of interoceptive signals in the brain to guide moral decision‐making.

## Author Contributions

**Shengbin Cui:** writing – original draft, investigation, formal analysis, data curation, visualization. **Tamami Nakano:** conceptualization, resource, funding acquisition, methodology, supervision, writing – reviewing and editing.

## Ethics Statement

This study was approved by the Research Ethics Committee of Osaka University and each participant signed a written informed consent.

## Conflicts of Interest

The authors declare no conflicts of interest.

## Supporting information


**Data S1:** Supporting Information.

**Data S2:** Supporting Information.

## Data Availability

The data that support the findings of this study are openly available in OSF at https://osf.io/agzfn/?view_only=5af0649706fa4ca79196ec2ad612e028.
