# Supplementary material for: Interoceptive Brain Processing Influences Moral Decision Making
Source: Hum Brain Mapp. 2024 Dec 25;45(18):e70108. doi: 10.1002/hbm.70108 (PMC11669002; doi:10.1002/hbm.70108)
Supplement: Supplementary file 1 — Data S1: Supporting Information. [file HBM-45-e70108-s002.pdf]

Odd Number: Personal
Even Number: Impersonal

1).
You and five other people are trapped in a burning building. There is only one emergency exit through which all of you could escape, but it is blocked by burning debris. Another injured person is about to crawl through a hole at the bottom of the exit door. You and the five people behind you do not have time to do the same.

If you use the injured person to break down the debris you will be able to escape. Doing this will certainly kill him, but it will save you and the five people behind you.

Do you break down the blockage by using the injured person so you and the five other people can escape?

> **Utilitarian Option:** Use the injured person to break down the debris, resulting in his death but allowing you and the five other people to escape.
>
> **Deontological Option:** Not using the injured person to break down the debris so that he can survive but causing no time for you and the five other people to escape and resulting in your deaths.

2).
You and five other people are trapped in a burning building. There is only one emergency exit through which all of you could escape, but it is blocked by burning debris. Another injured person is about to crawl through a hole at the bottom of the exit door. You and the five people behind you do not have time to do the same.

If you activate the emergency system, it will eliminate oxygen from the hallway which will put out the fire. However, it will leave the injured person without air. This will kill him, but you and the five people behind you will be saved.

Do you put out the fire by activating the emergency system, which will leave the injured without air, so you and the five other people can escape?

> **Utilitarian Option:** Activate the emergency system, resulting in the death of the injured person but allowing you and the five other people to be saved.
>
> **Deontological Option:** Do not activate the emergency system, causing the injured person to survive, but resulting in you and the other five people being unable to escape and your deaths.

3).
Enemy soldiers have taken over your village and will kill all civilians above the age of two. You and ten neighbors are hiding in two rooms of the cellar of a large house. You can hear the voices of soldiers who have come to search the house for valuables. Your

baby begins to cry loudly. The crying will attract the attention of the soldiers, who will spare your baby's life, but will kill you and the other refugees in both rooms.

If you put your hand over its mouth the crying will be absorbed, but your baby will not be able to breathe. This will kill it, but it will save you and the other ten neighbors.

Do you absorb the crying by putting your hand over your baby's mouth, which will leave it without air, so the soldiers won't find you and the ten neighbors?

> **Utilitarian Option:** Keep hand in place, resulting in the baby's death and sparing everyone else in the cellar.
>
> **Deontological Option:** Remove the hand from the baby's mouth, resulting in the death of everyone in the cellar except the baby.

4).
Enemy soldiers have taken over your village and will kill all civilians above the age of two. You and ten neighbors are hiding in two rooms of the cellar of a large house. You can hear the voices of soldiers who have come to search the house for valuables. Your baby begins to cry loudly. The crying will attract the attention of the soldiers, who will spare your baby's life, but will kill you and the other refugees in both rooms.

If you activate a noisy boiler, it will cushion the crying, but it will become uncomfortably hot. The heat will be mortal for your baby, but it will save you and the ten neighbors.

Do you cushion the crying by activating the noisy boiler which will asphyxiate the baby, so they won't find you and the ten neighbors?

> **Utilitarian Option:** Activate the noisy boiler, resulting in the baby's death and sparing everyone else in the cellar.
>
> **Deontological Option:** Do not activate the noisy boiler, resulting in the death of everyone in the cellar except the baby.

5).
You and ten divers are part of an U.N. team who is deactivating anti-ship mines from World War II. One team member has hurt himself and the blood in the water has attracted several sharks. You have an underwater rifle but only one harpoon and there are many sharks. The bleeding diver is swimming towards the last protective cage and will reach it before you and the others. The sharks, following the blood, are coming too close for you and the other divers to escape.

If you shoot at the injured diver this will kill him and the sharks will stop to eat him, but you and the ten divers will be saved.

Do you let the sharks eat the injured diver by shooting at him, so you and the other ten divers can reach the protective cage?

> **Utilitarian Option:** Shoot at the injured diver, resulting in the death of the diver and allowing you and 10 other divers to reach the protective cage.
>
> **Deontological Option:** Let sharks continue towards team of you and the 10 other divers, resulting in your death and sparing the one diver.

6).

You and ten divers are part of an U.N. team who is deactivating anti-ship mines from World War II. One team member has hurt himself and the blood in the water has attracted several sharks. You have an underwater rifle, but only one harpoon and there are many sharks. The bleeding diver is swimming towards the last protective cage and will reach it before you and the others. The sharks, following the blood, are coming too close for you and the other divers to escape.

If you detonate one of the mines by mean of a remote control, it will kill the injured diver and the sharks will stop to eat him. Then, you and the ten divers will be saved.

Do you let the sharks eat the injured diver by detonating the mine next to him with a remote control, so you and the other ten divers can reach the protective cage?

> **Utilitarian Option:** Detonate the mine with the remote control, resulting in the death of one diver and allowing you and 10 other divers to reach the protective cage.
>
> **Deontological Option:** Do not detonate the mine, causing the diver to reach the protective cage and resulting in the death of you and the 10 other divers.

7).

You are a Peace Corps health-worker who is volunteering in a rural African village. A man from a nearby village has been infected with the Ebola virus that is extremely contagious, incurable, and almost always deadly. Unexpectedly, this man is still alive which means he must be immune to the virus' deadly consequences. However, he wrongly believes that your health center can cure his disease completely. You see him approaching and you know that if he enters the village, he will pass the virus to 119 innocent people who, unlike him, will die.

If you shoot him with the gun of the health center, you will prevent him from spreading the virus to you and the rest of the village. This will kill him, but it will save you and the 119 inhabitants of the village.

Do you prevent the man from entering the village by shooting him, so he won't spread the virus to you and the 119 people of the village?

Utilitarian Option: Shoot at the man to prevent him from entering the village, resulting in his death, and sparing you and the other 119 people in the village.

Deontological Option: Do not shoot at the man, causing him alive but make the virus spread across the village and result in the death of you and 119 other people in the village.

8).

You are a Peace Corps health-worker who is volunteering in a rural African village. A man from a nearby village has been infected with the Ebola virus that is extremely contagious, incurable, and almost always deadly. Unexpectedly, this man is still alive, which means he must be immune to the virus' deadly consequences. However, he wrongly believes that your health center can cure his disease completely. You see him approaching and you know that if he enters the village, he will pass the virus to 119 innocent people who, unlike him, will die.

If you warn the village council, they will shoot him to prevent him from spreading the virus to you and the rest of the village. This will kill him, but it will save you and the 119 inhabitants of the village.

Do you prevent the man from entering the village by warning the village council so that they shoot him, so he won't spread the virus to you and the 119 people of the village?

Utilitarian Option: Warn the village council to prevent the man from entering the village, resulting in his death, and sparing you and 119 people in the village.

Deontological Option: Keep silent to the village council, causing the man alive but make the virus spread across the village and resulting in the death of you and 119 people in the village.

9).

You are a doctor alone in a rescue helicopter during a devastating flood. You have rescued eight critically injured people, and you are keeping them alive. Suddenly, the pilot notifies you of a problem in the engine and gives the order that the helicopter needs to release weight, or it will crash and you will all die. You cannot abandon the helicopter because the wounded need your help, and the heavy medical equipment is bolted down.

If you throw off one of the injured people, you will release enough weight to keep the helicopter in the air. This will kill him, but it will save you and the eight people on board.

Do you release weight by throwing one of the injured people off the helicopter, so you and the eight persons on board will be able to stay in the air?

Utilitarian Option: Throw one of the injured people off the helicopter to release weight. , resulting in the death of the man and enabling you and 8 people to stay on board in the air.

Deontological Option: Keep all of the people stay in the helicopter, causing the helicopter to crash and resulting in the death of all of you.

10).

You are a doctor alone in a rescue helicopter during a devastating flood. You have rescued eight critically injured people, and you are keeping them alive. Suddenly, the pilot notifies you of a problem in the engine and gives the order that the helicopter needs to release weight, or it will crash and you will all die. You cannot abandon the helicopter because the wounded need your help, and the heavy medical equipment is bolted down.

If you cut the cable by which the last rescued person is ascending, you will release enough weight to keep the helicopter in the air. This will kill him, but it will save you and the eight people on board.

Do you release weight by cutting the cable by which the last person is ascending, in order to keep you and the eight people on board in the air?

Utilitarian Option: Cut off the cable by which the last person is ascending to release weight, resulting in his death and enabling you and 8 people to stay on board in the air.

Deontological Option: Do not cut off the cable, causing the helicopter crash to the ground and resulting in the death of all of you.

11).

You are an engineer on the international space station ISS. Suddenly, a fire breaks out in the cargo bay. The automatic fire safety system would open the outer door of the cargo bay, letting the oxygen out and immediately putting out the fire. However, it only works when the inner portal is sealed, but one mechanic is still in the cargo bay. You realize that he doesn’t have the time to take off the bulky space suit and that he’ll get stuck when trying to get through the inner portal, causing the fire to spread and to kill you all.

If you push the mechanic back into the cargo bay, the portal will close activating the emergency system which will put out the fire. This will suck the mechanic into space and kill him, but it will save you and the other ten astronauts.

Do you put out the fire by manually sealing the inner portal, which will suck the mechanic into space, so the fire won’t reach you and the ten astronauts?

Utilitarian Option: Push the mechanic back into the cargo bay to activate the automatic fire safety system, causing the mechanic to be sucked into space and sparing you and 10 other astronauts.

Deontological Option: Do nothing, causing all of you die.

12).
You are an engineer on the international space station ISS. Suddenly, a fire breaks out in the cargo bay. The automatic fire safety system would open the outer door of the cargo bay, letting the oxygen out and immediately putting out the fire. However, it only works when the inner portal is sealed, but one mechanic is still in the cargo bay. You realize that he doesn't have the time to take off the bulky space suit and that he'll get stuck when trying to get through the inner portal, causing the fire to spread and to kill you all.

If you push a button before the mechanic reaches the inner portal, the portal will close activating the emergency system which will put out the fire. This will suck the mechanic into space and kill him, but it will save you and the remaining ten astronauts.

Do you put out the fire pushing the button which seals the inner portal, sucking the mechanic into space, so the fire won't reach you and the ten astronauts?

Utilitarian Option: Push the button to close the inner portal and activate the emergency system to put out the fire, causing the mechanic to be sucked into space and sparing you and 10 other astronauts.

Deontological Option: Do nothing, causing the death of all of you because of the fire.

13).
You are an inspector of a nuclear power plant. The plant foreman and you are visiting the facility when one of the supply conduits overheats. The emergency coolant system fails to activate, and a chain reaction which will result in a nuclear meltdown is about to start. This will release lethal radiation into the nearby town, killing thousands of people.

If you push the foreman onto the conduits, it will release enough heat to prevent the nuclear chain reaction. This will kill him, but it will save you and the thousands of town inhabitants.

Do you cool down the conduits by pushing the foreman down on them, so the nuclear chain reaction will be stopped in time for you and the thousands of people?

Utilitarian Option: Push the foreman onto the conduits to stop the reaction, resulting in the death of the foreman and saving you and the thousands of the town inhabitants.

Deontological Option: Do not push the foreman and let the reaction to continue, causing you and the thousands of the town inhabitants to die.

14).

You are an inspector of a nuclear power plant. The plant foreman and you are visiting the facility when one of the supply conduits overheats. The emergency coolant system fails to activate, and a chain reaction which will result in a nuclear meltdown is about to start. This will release lethal radiation into the nearby town, killing thousands of people.

If you insert liquid nitrogen manually into the supply chamber it will release enough heat to prevent the nuclear chain reaction. This will kill one employee, who is trapped in the supply chamber, but it will save you and the thousands of town inhabitants.

Do you release the heat inserting liquid nitrogen into the supply chamber where the employee is trapped, so the nuclear chain reaction will be stopped in time for you and the thousands of people?

Utilitarian Option: Insert liquid nitrogen manually into the supply chamber to release heat, causing the foreman to die and saving you and the thousands of the town inhabitants.

Deontological Option: Do not insert liquid nitrogen into the supply chamber, causing the reaction to continue and resulting in the death of you and the thousands of town inhabitants.

15).

You are a construction worker. You and your crew are standing on some scaffolding, working on a skyscraper. Suddenly, the scaffolding collapses partially. You and several others are hanging on to a dangling crossbar, but it cannot hold the weight of all of you. A worker next to you slips off the crossbar and grabs your one free arm. However, you realize that the entire structure is about to give way.

If you repeatedly kick your coworker in the stomach and chest until he lets, go of your hand and falls, you will remove just enough weight so the rest of you can make it to safety before the scaffolding collapses entirely. This will kill your colleague, but it will safe you and the others.

Do you remove weight by hitting this colleague until he lets go of your arm and falls, so you and the others can continue to hold on to the structure?

Utilitarian Option: Hit the colleague until he falls to remove weight, resulting in his death and sparing you and other colleagues.

Deontological Option: Let the colleague catch your arm, causing the scaffolding to collapse and resulting in your and others' death.

16).

You are a construction worker. You and your crew are standing on some scaffolding, working on a skyscraper. Suddenly, the scaffolding collapses partially. You and several others are hanging on to a dangling crossbar, but it cannot hold the weight of all of you. The worker next to you slips off the crossbar and catches himself on another portion of the scaffolding. The entire structure is about to give way.

If you pull out a latch that will detach the section of scaffolding with your co-worker on it, this will remove just enough weight that the rest of you can make it to safety before the scaffolding collapses entirely. This will kill him but save you and the other five coworkers.

Do you remove weight by pulling out the latch so your co-worker falls off the crossbar, so you and the others can continue to hold on to the structure?

> **Utilitarian Option:** Pull out the latch to release weight, resulting in the death of the co-worker and sparing you and other colleagues.
>
> **Deontological Option:** Let the co-worker to catch the scaffolding, causing the death of you and other colleagues.

17).

You are the bus driver of a kindergarten trip to the zoo. On the way, faulty tires cause the bus to plunge off a bridge and into a roaring river. You and three of the children are still in the sinking bus. The rest has already been swept away by the river. You grab the two children next to you and begin to swim towards the exit. The third child latches onto your leg. You feel that you are not strong enough to fight against the current and swim with all three children.

If you shake off the third child you will be able to fight against the current, but the child dropped behind will drown. This will kill him, but it will save you and the other two children.

Do you fight against the current shaking off the child, which will make him to drown, so you and the other two children can reach the surface?

> **Utilitarian Option:** Shake off the child to fight against the current, resulting in the death of the child and sparing you and the other two children.
>
> **Deontological Option:** Do not shake off the child, causing you and 3 children drawn to the death.

18).

You are the bus driver of a kindergarten trip to the zoo. On the way, faulty tires cause the bus to plunge off a bridge and into a roaring river. You and three of the children are still in the sinking bus. The rest has already been swept away by the river. You grab the

two children next to you and begin to swim towards the exit, but bulky seats are blocking it.

If you pull the seats away you will free the exit, but you will block the corridor and the third child will be trapped, causing him to drown. This will kill him, but it will save you and the other two children.

Do you free the exit putting the seats in the corridor, which will trap the third child, so you and the other two children can reach the surface?

> **Utilitarian Option:** Pull the seats away to free the exit, causing the third child stuck and drown to death and saving you and other two children.
>
> **Deontological Option:** Do not pull the seats away, causing you and 3 children drown to the death.

19).
A viral epidemic has spread across the globe killing thousands of people. You are a medical researcher and have developed two substances in your laboratory. Due to a mistake both are labeled as vaccine. One of them is indeed the vaccine, which could save thousands of lives. The other one is lethal.

If you inject the substances to your two lab assistants without knowing which substance is which, you will be able to identify the vaccine. One of the assistants will die, but you will be able to save thousands of lives with your vaccine.

Do you identify the vaccine injecting these two substances into your two lab assistants which will poison one of them, so thousands of people can be vaccinated?

> **Utilitarian Option:** Inject the substances into the two lab assistants to identity the vaccine, resulting in the death of one of them and sparing thousands of people.
>
> **Deontological Option:** Do not inject the substances to identify the vaccine, causing the death of thousands of infected people.

20).
A viral epidemic has spread across the globe killing thousands of people. You are a medical researcher and have developed two substances in your laboratory. Due to a mistake both are labeled as vaccine. One of them is indeed the vaccine, which could save thousands of lives. The other one is lethal.

If you allow a nurse to inject the two substances to two patients, you will be able to identify the vaccine. One of the patients will die, but you will be able to save thousands of lives with the vaccine.

Do you identify the vaccine allowing the nurse to inject the two substances to two patients which will poison one of them, so thousands of people can be vaccinated?

> **Utilitarian Option:** Let the nurse to inject the substances into the two patients to identify the vaccine, resulting in the death of one of them and sparing thousands of people.
>
> **Deontological Option:** Do not inject the substances into the patients, causing them alive and resulting in the death of thousands of infected people.

21).
A runaway trolley is speeding down the tracks towards five workmen who will be killed if the trolley continues on its present course. You are standing next to the tracks, but you are too far away to warn them. Next to you there is a very large stranger.

If you push the large stranger onto the tracks, the trolley will slide off the tracks and won't continue its course towards the workmen. This will kill the stranger, but you will save the five workmen.

Do you cause the trolley to derail pushing the stranger onto the tracks, so the trolley does not reach the five workmen?

> **Utilitarian Option:** Push the large stranger onto the tracks to stop the trolley, resulting in the death of the stranger and sparing the five workmen.
>
> **Deontological Option:** Do nothing to stop the trolley, causing the death of five workmen.

22).
A runaway trolley is speeding down the tracks towards five workmen who will be killed if the trolley continues on its present course. You are standing next to the tracks, but you are too far away to warn them. Next to you there is a control switch that can redirect the trolley onto a different track, where only one workman is working.

If you flip the control switch it will divert the trolley onto the track with one workman and will not continue its course towards the five workmen. This will kill this workman, but it will save the other five.

Do you divert the trolley by flipping the control switch, which will cause the trolley to run over one workman, so the trolley does not reach the five workmen?

> **Utilitarian Option:** Flip the control switch to divert the trolly onto another track, causing the death of one workman and sparing five workmen.
>
> **Deontological Option:** Do not divert the trolley with the control switch, causing the death of five workmen.

23).

You and a fellow researcher have discovered a powerful new energy source that is cheap, safe, and clean. It has the potential to put an end to pollution and poverty in the world However, your colleague wants to sell the discovery and you know that he is planning to contact the potential buyers today. You also know for sure that the latter will use the invention as a deadly weapon, but your colleague doesn't believe you.

If you poison your colleague with a common poison available in your lab, you will avoid having the discovery fall into the wrong hands. The poison will kill your colleague, but you will save thousands of people.

Do you prevent the sale of the discovery by poisoning your colleague, so the source of energy can't be used as a weapon against thousands of people?

> **Utilitarian Option:** Poison the colleague with the poison to stop your colleague, causing him to death and preventing the source of energy from being used as a weapon and sparing thousands of people.
>
> **Deontological Option:** Do not poison your colleague, causing the source of energy to be used as a weapon against thousands of people.

24).

You and a fellow researcher have discovered a powerful new energy source that is cheap, safe, and clean. It has the potential to put an end to pollution and poverty in the world However, your colleague wants to sell the discovery and you know that he is planning to contact the potential buyers by email today. You also know for sure that the latter will use the invention as a deadly weapon, but your colleague doesn't believe you.

If you release a flammable gas in the lab, it will cause an explosion when your colleague turns on his computer. This will kill him, but you will save thousands of people.

Do you cause the computer of your colleague to explode releasing the flammable gas, in order to prevent the discovery being used as a weapon against thousands of people?

> **Utilitarian Option:** Release a flammable gas into the lab, causing an explosion to make your colleague to die and sparing thousands of people.
>
> **Deontological Option:** Do nothing to your colleague, causing the source of energy to be used as a weapon against thousands of people.

25).

You are the leader of a small group of soldiers. You are on your way back from a mission in enemy territory when one of your men steps in a trap that catches his leg, injuring him badly. You cannot free him without killing him, but if you leave him behind, enemy troops will torture him until he reveals the position of an important allied base camp. After that the soldiers will attack this camp and kill the 10 soldiers of the platoon

there. The soldier begs you not to leave him behind, but the enemy troops are approaching, and it is dangerous for you and your men to stay because you don't have any more ammunition.

If you stab the soldier yourself, you will impede that he'll be tortured and reveal the information which leads to the death of the allied platoon. This will kill him, but you will save the ten soldiers of the platoon.

Do you impede that the trapped soldier will be tortured by stabbing him to death, so he can't reveal the location of the ten allied troop members?

> **Utilitarian Option:** Stab the soldier to impede his being tortured and information being revealed, causing him to death and saving other ten soldiers.
>
> **Deontological Option:** Do not kill the trapped soldier, causing the information to get exposed and resulting in the death of the ten allied troop members.

26).

You are the leader of a small group of soldiers. You are on your way back from a mission in enemy territory when one of your men steps in a trap that catches his leg, injuring him badly. You cannot free him without killing him, but if you leave him behind, enemy troops will torture him until he reveals the position of an important allied base camp. After that the soldiers will attack this camp and kill the 10 soldiers of the platoon there. The soldier begs you not to leave him behind, but the enemy troops are approaching, and it is dangerous for you and your men to stay because you don't have more ammunition.

If you signalize the position of the trapped soldier with an illumination rocket, the region will be bombed. Thus, you will avoid that the soldier is tortured and reveals the information which will lead to the death of the allied troop. This will kill him, but you will save the ten soldiers of the allied platoon.

Do you impede that the trapped soldier is tortured by ordering the bombing of that region, so he can't reveal the location of the ten allied troop members?

> **Utilitarian Option:** Signal the position of the trapped soldier with an illumination rocket, causing the region to be bombed and resulting in the death of the soldier and sparing other ten soldiers.
>
> **Deontological Option:** Not get the region to be bombed, causing the information to get exposed and resulting in the death of the ten allied troop members.

27).

You are on the night watch in a hospital. A chemical product has been spilled in a room with six patients. This chemical is highly toxic because once exposed to oxygen it evaporates and transforms into a poisonous gas which will kill all the patients in the

room. You cannot wipe it away with conventional towels or wiping clothes. However, human skin absorbs it swiftly.

If you place one of the patients on the chemical, you will prevent it from evaporating. This will kill this patient, but you will save the other five patients in the room.

Do you prevent the toxic evaporation placing one of the patients on the chemical, so the other five patients won't be poisoned?

> **Utilitarian Option:** Place the patient on the chemical to stop evaporation, causing the death of the patient and sparing the other 5 patients in the room.
>
> **Deontological Option:** Let the chemical to evaporate, causing the other 5 patients in the room to die.

28).

You are on the night watch in a hospital. A chemical product has been spilled in a room with five patients. This chemical is highly toxic because once exposed to oxygen it evaporates and transforms into a poisonous gas which will kill all the patients in the room. You cannot wipe it away with conventional towels or wiping clothes.

If you activate the ventilation system, the poisonous gas will be taken out of the room. However, it will be transferred into a room upstairs with one patient, whom you won't be able to evacuate in time. This will kill this patient, but you will save the other five.

Do you divert the gas out of the room by activating the ventilation system, causing it to be transferred to the room with one patient, so the other five patients won't be poisoned?

> **Utilitarian Option:** Activate the ventilation system to take the poisonous gas out of the room, causing the patient in a room upstairs to die due to transferred gas and sparing the other 5 patients.
>
> **Deontological Option:** Let the chemical to transform into poisonous gas, causing the death of the 5 patients in the room.

29).

You are on a rowboat with a tour guide on a lake in Alaska. Nearby, three children have fallen off their boat and are in danger of freezing to death. You rescue the children, but one of them is very heavy and you realize that if you keep him on board your boat will sink, and all will die. Neither you nor the guide can leave the boat because you are rowing, and the guide is giving CPR to one of the children.
If you throw the heavy child off the boat, you will be able to reach the shore. This will kill this child, but you will save the other two.
Do you get rid of load by throwing that one child overboard, so you can reach the shore with the other two children?

Utilitarian Option: Throw the heavy child off the boat to release weight, causing the child to death and allowing you and other 3 people reach the shore.

Deontological Option: Let the heavy child stay in the boat, causing the boat sink and resulting the death of all of you.

30).

You are on a rowboat with a tour guide on a lake in Alaska. Nearby, three children have fallen off their boat and are in danger of freezing to death. After you rescue two of the children, you realize that the weight of the third child would cause your boat to sink and all would die. Neither you nor the guide can leave the boat because you are rowing, and the guide is giving CPR to one of the children.
If you row to the shore leaving the third child behind in the water, you will prevent your boat from sinking. This will kill the child left behind, but you will save the other two.
Do you prevent your boot from sinking by leaving the third child behind, which will cause him to die, so that you can reach the shore with the other two children?

Utilitarian Option: Leave the third child in the water, causing the child to drown to the death and allowing you and other 3 people to reach the shore.

Deontological Option: Let the third child get into the boad, causing the boad to sink and resulting in the death of all of you.

31).

You are an electrician hired to fix an electrical problem in the home of a very wealthy tycoon who is terminally ill of cancer. Recently, it has been announced that upon his death, his enormous fortune will be donated to the local children's hospital. You know that there are 109 children at this hospital who could be saved with this money, but who cannot wait much longer before they die.

If you suffocate the tycoon while he is sleeping by covering his nose and mouth with your gloved hands, this essential donation for the children will arrive in time. This will kill the tycoon, but you will save the lives of 109 children.

Do you speed the donation by suffocating this man, so that the donation will get to the 109 ill children in time?

Utilitarian Option: Suffocate the tycoon with your gloved hands, causing the tycoon to die and allowing the 109 children to receive the donation in time.

Deontological Option: Do not suffocate the tycoon, causing the donation to be late and resulting in the death of 109 children.

32).

You are an electrician hired to fix an electrical problem in the home of a very wealthy tycoon who is terminally ill of cancer. Recently, it has been announced that upon his

death, his enormous fortune will be donated to the local children's hospital. You know that there are 112 children at this hospital who could be saved with this money, but who cannot wait much longer before they die.

If you short-circuit the cables instead of fixing the problem, the tycoon's life support equipment will fail and this essential donation for the children will arrive in time. This will kill the tycoon, but you will save the lives of 112 children.

Do you anticipate the donation by causing the tycoon's life support equipment to fail, so that the donation gets to the 112 ill children in time?

> **Utilitarian Option:** Short-circuit the cables to destroy the life support equipment, causing the tycoon to die and allowing the 109 children to receive the donation in time.
>
> **Deontological Option:** Fix the electrical problem, causing the donation to be late and resulting in the death of 109 children.

33).

You are a doctor. One of your patients, who is HIV positive, is about to receive a blood transfusion before being released from the hospital. He has told you, in the confidence of your doctor-patient relationship, that he intends to infect as many people as possible starting that evening, after leaving the hospital. There is no legal way to stop him, because the police cannot arrest him based on protected information.

If you contaminate his medication by putting an untraceable poison in it, you will prevent him from infecting others. This will kill him, but you will save many.

Do you prevent the transmission of AIDS by poisoning the patient, so he won't be able to infect other people?

> **Utilitarian Option:** Contaminate patient's medication, resulting in his death and sparing the lives of the many he will infect.
>
> **Deontological Option:** Leave medication intact, sparing patient from more immediate death and resulting in the deaths of many he will infect.

34).

You are a doctor. One of your patients, who is HIV positive, is about to receive a blood transfusion before being released from the hospital. He has told you, in the confidence of your doctor-patient relationship, that he intends to infect as many people as possible starting that evening, after leaving the hospital. There is no legal way to stop him, because the police cannot arrest him based on protected information.

If you change his blood-type in the hospital's database, he will receive the wrong blood and you will prevent him from infecting many people. This will kill him, but you will save many.

Do you prevent the transmission of AIDS by changing the blood-type of the patient, so he won't be able to infect other people?

> **Utilitarian Option:** Modify his blood type in the database, causing him to die because of the wrong blood he got received and sparing the lives of the many he will infect.
>
> **Deontological Option:** Do not change the database, sparing patient from more immediate death and resulting in the death of many he will infect.

35).
You are negotiating with a psychologically ill and armed man on the roof of a building. Down below there is a crowd of people at the city's art and music festival. He wants to shoot people in the crowd, and you have tried but cannot talk him out of it. The crowd does not realize the threat and you have no way to warn them. From where he is standing, he could easily kill a dozen people, not counting those who would be injured or killed by the resulting chaos.

If you run over and shove him off the roof of the building, he won't be able to shoot at people in the crowd. This will kill him, but you will save the lives of many.

Do you prevent the chaos by shoving the man off the roof, so he won't shoot at people in the crowd?

> **Utilitarian Option:** Run over and shove the man off the roof of the building to prevent him from shooting, causing him to die and sparing the lives of many.
>
> **Deontological Option:** Do not run over, causing him alive and resulting in the death of many because of his shooting.

36).
You are negotiating with a psychologically ill and armed man on the roof of a building. Down below there is a crowd of people at the city's art and music festival. He wants to shoot people in the crowd, and you have tried but cannot talk him out of it. The crowd does not realize the threat and you have no way to warn them. From where he is standing, he could easily kill a dozen of people, not counting those who would be injured or killed by the resulting chaos.

If you inform the police that the negotiation has failed, they will shoot him and prevent the shooting at the crowd. This will kill him, but you will save the lives of many.

Do you prevent the chaos by letting the police shoot him, so he won't shoot at people in the crowd?

> **Utilitarian Option:** Inform the police and let them shoot the man, causing him to die and saving the lives of many.
>
> **Deontological Option:** Do not notify the police, causing him alive and resulting in the death of many because of his shooting.
