# Supplementary material for: Interoceptive Brain Processing Influences Moral Decision Making
Source: Hum Brain Mapp. 2024 Dec 25;45(18):e70108. doi: 10.1002/hbm.70108 (PMC11669002; doi:10.1002/hbm.70108)
Supplement: Supplementary file 2 — Data S2: Supporting Information. [file HBM-45-e70108-s001.pdf]

Supplementary Figures

1.

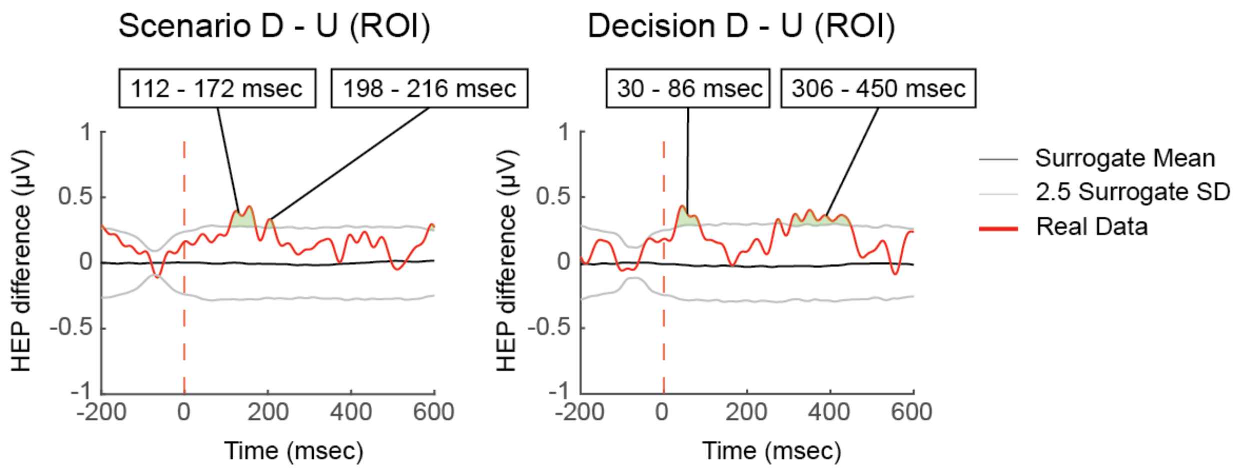

Figure 1. Comparison of HEP differences between real data and surrogate data. **Left panel**. The difference in HEPs between deontological and utilitarian decisions (red line) during the Scenario Phase was compared with the same metric derived from 1,000 surrogate data. The black line represents the averaged surrogate HEP difference. The green shaded area indicated the time during which real data surpasses 2.5 surrogate SD (grey lines). **Right panel**. The difference in HEPs between deontological and utilitarian decisions (red line) during the Decision Phase against surrogate data. The green shaded area represents the time during which real data surpasses 2.5 surrogate SD.

2.

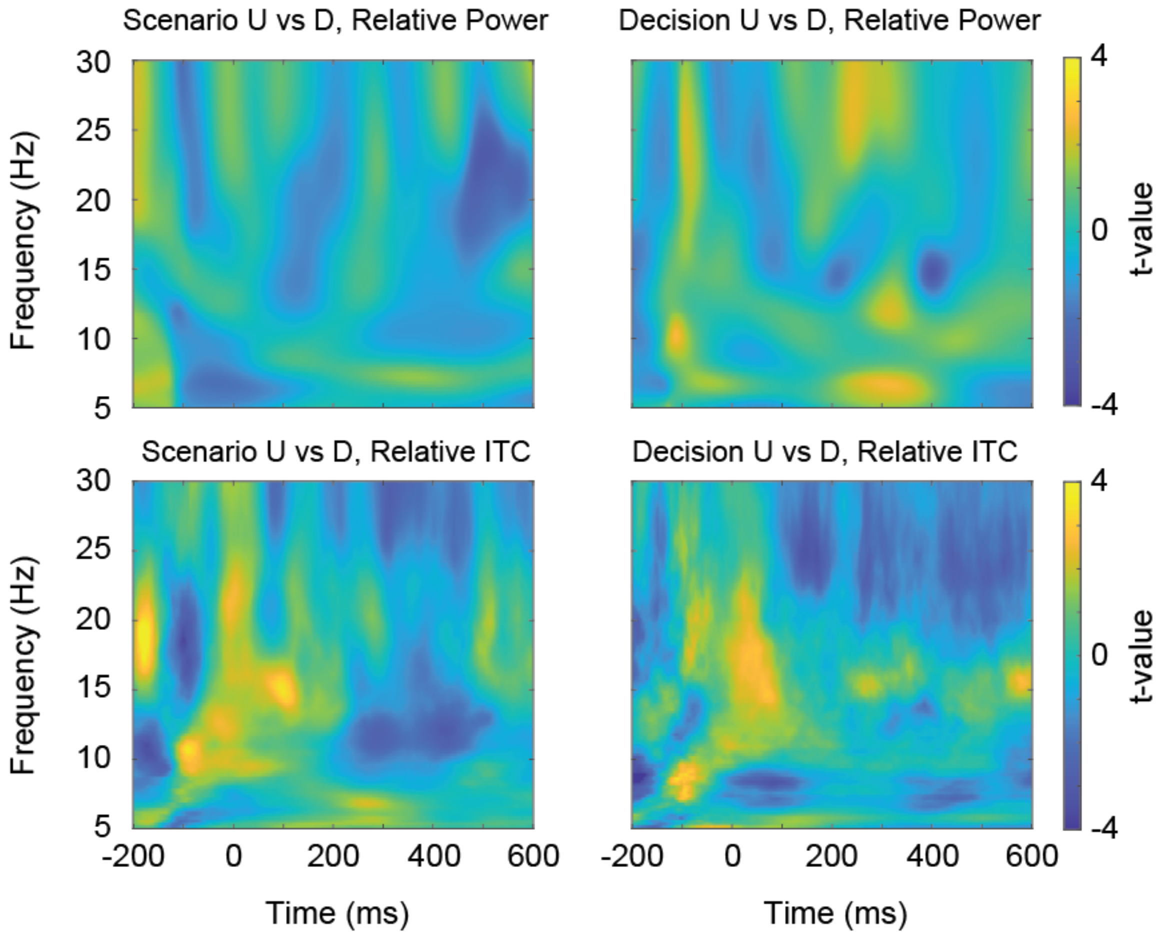

Figure 2. The result of cluster-based permutation tests on time-frequency representations of averaged HEPs over the electrodes with significant difference during the Scenario and Decision phases. No cluster with significant difference was observed in relative power between utilitarian and deontological decisions during the Scenario (**top left**) and Decision phases (**top right**). Also, no cluster with significant difference was observed in relative ITC during the Scenario (**bottom left**) and Decision phases (**bottom right**) between utilitarian and deontological decisions.
